# Aptamers Selected for Recognizing Amyloid β-Protein—A Case for Cautious Optimism

**DOI:** 10.3390/ijms19030668

**Published:** 2018-02-27

**Authors:** Farid Rahimi

**Affiliations:** Division of Biomedical Science and Biochemistry, Research School of Biology, The Australian National University, Canberra, ACT 2601, Australia; farid.rahimi@anu.edu.au or z2170549@zmail.unsw.edu.au; Tel.: +61-2-6125-2851

**Keywords:** Alzheimer disease, amyloid β-protein, antibodies, cross-reactions, nucleotide aptamers, oligonucleotide ligands, systematic evolution of ligands by exponential enrichment, specificity, therapeutics

## Abstract

Aptamers are versatile oligonucleotide ligands used for molecular recognition of diverse targets. However, application of aptamers to the field of amyloid β-protein (Aβ) has been limited so far. Aβ is an intrinsically disordered protein that exists in a dynamic conformational equilibrium, presenting time-dependent ensembles of short-lived, metastable structures and assemblies that have been generally difficult to isolate and characterize. Moreover, despite understanding of potential physiological roles of Aβ, this peptide has been linked to the pathogenesis of Alzheimer disease, and its pathogenic roles remain controversial. Accumulated scientific evidence thus far highlights undesirable or nonspecific interactions between selected aptamers and different Aβ assemblies likely due to the metastable nature of Aβ or inherent affinity of RNA oligonucleotides to β-sheet-rich fibrillar structures of amyloidogenic proteins. Accordingly, lessons drawn from Aβ–aptamer studies emphasize that purity and uniformity of the protein target and rigorous characterization of aptamers’ specificity are important for realizing and garnering the full potential of aptamers selected for recognizing Aβ or other intrinsically disordered proteins. This review summarizes studies of aptamers selected for recognizing different Aβ assemblies and highlights controversies, difficulties, and limitations of such studies.

## 1. Introduction

**Figure 1:**
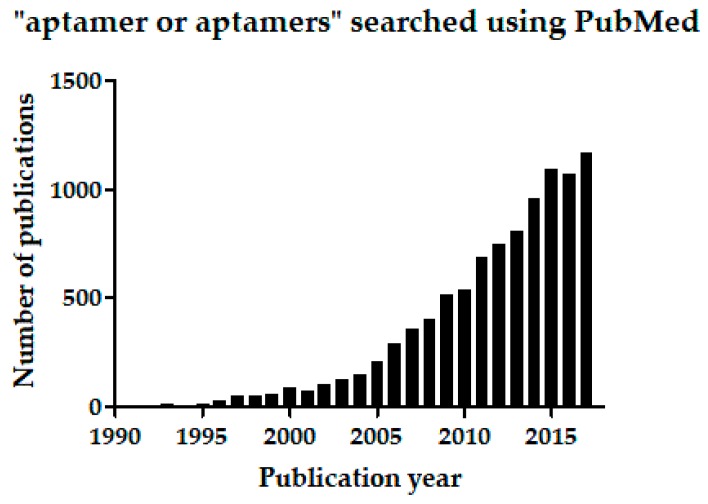
The phrase “aptamer or aptamers” was used as the search term on Pubmed. The number of publications are plotted per publication year.

Specifically detecting or recognizing targets of interest—by molecular recognition—is fundamental in many medical and scientific applications. Conventionally, antibodies have been used for detecting antigenic targets, which may include large proteins, small peptides, polysaccharides, lipids, or nucleic acids. Antibodies have been essential for diagnostic or routine clinical assays and immunotherapeutic applications, and in important techniques such as immunohistochemistry, immunoprecipitation, enzyme-linked immunosorbent assay (ELISA), and western blotting. Besides antibodies, however, nucleotide aptamers (oligonucleotide ligands) have emerged since 1990 and progressed rapidly as alternative molecular-recognition tools, offering many useful and novel scientific applications [1,2,3,4,5,6]. So far, US Food and Drug Administration (FDA) has approved one aptamer drug, Macugen^®^ or generically pegaptanib (Pfizer Pharmaceuticals Group, New York, NY, USA) [7,8], and reportedly, some additional ten aptamers have undergone clinical trials for treating various conditions, including macular degeneration, coagulation, cancer, and inflammation [5,6]. The continually increasing number of reports on aptamers published since the initial 1990 publications [9,10,11,12] also vouches for the rapid progress of aptamer science. Searching the MEDLINE database through PubMed for the phrase “aptamer or aptamers” returns 328 reports published in the first decade of aptamer research. The same search returns 9459 reports collectively from 2001 to the end of December 2017 (Figure 1). (Related to aptamers, spiegelmers also are synthetic ligand-binding oligonucleotides, but spiegelmers comprise non-natural l-nucleotides [13]).

Importantly, aptamers offer additional advantages that antibodies do not (aptamer advantages and disadvantages have been extensively compared to those of antibodies elsewhere [5]). For example, aptamers effectively cause low or no immunogenicity and can be “selected” for many diverse molecules, including toxic compounds, for which generating antibodies in vivo would likely be impossible. Individual aptamers always interact with a single “aptatope”, whereas antibodies could be monoclonal or polyclonal. Moreover, an aptamer’s selectivity and specificity for a particular target’s aptatope can potentially be tested during the aptamer-selection process in vitro. An antibody’s target specificity, however, cannot be ensured because antibodies are generated in vivo, and their capability to distinguish between specific antigenic and less structured epitopes (e.g., polyclonal antibodies) or between conformational epitopes (e.g., monoclonal antibodies) and their closely related molecular structures is determined post facto.

Conformational specificity of antibodies or that of aptamers becomes particularly crucial for targeting intrinsically disordered proteins (IDPs). Many amyloidogenic proteins belong to IDPs [14,15,16]. IDPs are heterogeneous proteins that exist in a dynamic conformational equilibrium under physiological conditions and present a time-dependent ensemble of short-lived structures that are likely difficult to isolate or stabilize. This conformational behavior, as well as homooligomerization and fibrillization, characterize amyloid β-protein (Aβ) [17,18], a metastable, amyloidogenic IDP that is controversially linked to pathogenesis of Alzheimer disease (AD). Some antibodies generated against a certain assembly of Aβ reportedly cross-react with other assemblies of this peptide. Similarly, polyclonal antibodies generated against oligomeric or fibrillar Aβ may cross-react with structurally similar assemblies of other IDPs unrelated to Aβ [19,20]. Therefore, specificities of some Aβ antibodies have been unconvincing and disputed [21,22], and studies using such disputed antibodies should be revisited and interpreted carefully. Akin to reports using controversial antibodies or studies using insufficiently characterized antibodies against Aβ, collective evidence on aptamers selected for reacting with Aβ highlights undesirable or unexpected interactions despite implementing strict selection experiments. Such studies should also be reconsidered and reviewed carefully.

This review discusses controversies and methodological limitations of using and characterizing aptamers selected for recognizing, mainly, Aβ, while alluding to some other relevant studies of Aβ-unrelated IDPs. To set the scene and before summarizing aptamer studies relevant to Aβ (in Section 4), I briefly introduce this peptide in Section 2 and write about aptamers and the systematic evolution of ligands by exponential enrichment (SELEX) in Section 3. In Section 5, I highlight the shortcomings of sodium dodecyl sulfate–polyacrylamide gel electrophoresis (SDS–PAGE) in characterizing Aβ assemblies and in assessing aptamer/antibody specificities for such assemblies. Finally, I sum up with contextual conclusions.

## 2. Amyloid β-Protein and Alzheimer Disease

Aβ is produced mainly as Aβ40 or Aβ42 (respectively comprising 40 or 42 amino acid residues) from the amyloid β-protein precursor (APP), when APP is sequentially cleaved by β-secretase and γ-secretase [23,24]. Aβ is produced in its monomeric form as a normal, physiologically relevant peptide [25,26,27,28,29,30,31,32,33,34], but it has been studied profusely in pathogenic, protein-misfolding contexts underlying AD. Aβ’s normal functions and its cytotoxic effects may be regulated by its local concentration; for example, picomolar amounts of synthetic Aβ42 reportedly enhance long-term potentiation and hippocampus-dependent memory in mice, whereas nanomolar levels of the same peptide yield the opposite effects [31]. Long-term potentiation is an electrophysiological paradigm for learning and memory composition, but its role in this capacity has been debated [35]. The above concentration-dependent effects are not unique to Aβ function in the brain. As an aside, S100B, a calcium-binding protein abundant in the brain and implicated in AD pathogenesis [36,37], exerts neurotrophic or neurotoxic effects at nanomolar or micromolar concentrations, respectively [38].

Aβ’s pathogenic premises in AD have been based on the classical amyloid cascade hypothesis [39] and its contemporary, revised version [40,41,42]. The classical amyloid cascade hypothesis posited that overproduction and deposition of Aβ fibrils in amyloid plaques, the pathological hallmarks of AD, cause AD and that formation of neurofibrillary tangles (the other AD hallmark), cell loss, vascular damage, and dementia are direct results of Aβ deposition [39]. The contemporary/revised version of the amyloid cascade hypothesis—the oligomer cascade hypothesis [43]—gives primacy to the neurotoxic and synaptotoxic effects of soluble, prefibrillar oligomeric Aβ assemblies in AD pathogenesis [42]. Therefore, many trials have attempted to target Aβ for therapeutic or diagnostic purposes [44,45]. However, the two cascade hypotheses have been consistently debated and challenged [46,47,48,49]. Furthermore, since the original observations that Aβ is a major component of plaques in AD-afflicted brains [50,51,52,53,54], and that the plaques contain fibrillar, β-sheet-rich Aβ [55,56], and since introduction of the revised hypothesis [40,41], diverse but elusive structural assemblies of Aβ have been described and studied profusely in vitro [43,57,58,59,60], adding to the complexity of Aβ-oligomer literature. These assemblies have been studied or described structurally, functionally, or both, but their interrelationships, and more importantly, their relevance to AD pathogenesis and progression are still enigmatic [57,60] particularly because their undisputed identification or characterization in vivo has been challenging [47].

Importantly, the AD plaque core contains not only Aβ—as thought [61,62,63]—but also other potential products of APP processing [22,64], other proteinaceous and nonproteinaceous components, including glycosaminoglycan, collagen, lipids, metal ions, reactive oxygen species, inflammatory proteins, and nucleic acids [65,66,67,68,69,70,71,72,73,74,75,76,77]. These observations suggest that diverse detrimental mechanisms, other than or additional to misfolding or deposition of Aβ, may underlie AD pathogenesis or progression. These mechanisms include disruption of cellular metabolism [78,79], deregulation of synapse structure and function [80], membrane damage [81], ionic imbalance [82], oxidative stress [83], inflammatory stress [84,85,86], and apoptotic [78] or other cytotoxic effects. Aβ by itself is unlikely to be underlying AD pathogenesis or progression. This is corroborated by the failure or discontinuation of some high-profile clinical trials designed based on the amyloid cascade hypothesis [47], repudiating the notion that Aβ is central to AD pathogenesis. It is likely that targeting of Aβ by some means may disrupt its physiological roles and may not be effective therapeutically in humans [47]. Although this may be an unresolved controversy, the physiological roles of Aβ should be considered when designing Aβ-targeting therapeutics.

## 3. Aptamers and Systematic Evolution of Ligands by Exponential Enrichment 

Aptamers for a target are selected from a pool of random nucleotides by a combinatorial, in vitro molecular-evolution technique termed—SELEX [9,10,12]. Two groups first used SELEX to select highly avid and specific RNA aptamers for particular targets, including organic dyes [10] and bacteriophage T4 DNA polymerase [12,87]. Since then, aptamers have been selected for a variety of targets, including metal ions [88], organic molecules [89], amino acids [90,91,92], viral nucleic acid components [93,94,95,96], peptides [97], proteins [5,6,98], drugs [99,100,101], macromolecules [102,103,104,105], cells [106,107], and pathogens [108,109,110,111,112,113].

SELEX is an iterative process that enables selecting and amplifying a specific property (e.g., avid binding for aptamers, or enzymatic activity for ribozymes or DNAzymes) from a large pool of oligonucleotide sequences, similar to a miniature Darwinian evolution [6,114]. A typical SELEX experiment includes repeated rounds of (1) incubating a library of random oligonucleotide sequences (~10^13^–10^15^ unique sequence in a naïve, unselected pool) with a target molecule; (2) separating target-bound sequences from unbound sequences; (3) dissociating the oligonucleotide–target complexes; and (4) amplifying, identifying, and sequencing the resultant, selected oligonucleotide pool, which contains potentially specific and avid aptamers for the target [114]. Repeated rounds of SELEX are driven by affinity to the target and by competition amongst random sequences. Preselection (negative SELEX) and counter-SELEX (subtractive SELEX) can be interspersed between certain rounds of SELEX respectively to remove sequences that nonspecifically bind to the partitioning matrix or those that bind to molecules closely similar to the actual target [114]. The final oligonucleotide pool becomes enriched with a relatively small number of sequences that, in case of aptamers, bind the target avidly and hopefully specifically. (In case of ribozymes and DNAzymes, sequences with desired catalytic activities are enriched [115,116].) The resultant aptamers can be amplified by polymerase chain reaction (PCR), products of which can then be cloned and sequenced to identify the best binding sequences. Finally, binding affinities, specificities, and cross-reactivity of aptamers are determined [117], and post-SELEX modifications are applied to improve affinity, specificity, stability, pharmacokinetics, or bioavailability of aptamers [114,118].

Since its inception [10,12,87], many variations of SELEX have been developed and used, achieving targeted and specific outcomes [119,120], and SELEX has been optimized and extended to isolation of RNA, single-stranded DNA, or modified versions thereof.

The discriminatory power and specificity of aptamers in some cases are surprisingly high. Aptamers reportedly can discriminate targets based on subtle chemical differences e.g., presence or absence of a methyl/hydroxyl group or chirality (*R* vs. *S* enantiomer). For example, a theophylline-specific aptamer distinguishes it from caffeine—which differs from theophylline by only one methyl group—at least ten-fold more efficiently than an antibody generated for this purpose [121]. Similarly, an enantioselective, modified DNA aptamer could distinguish (*R*)-thalidomide from (*S*)-thalidomide [122]. Such high levels of aptamer specificity result from the selective pressure achieved by counter-SELEX (subtractive SELEX) [121].

Selecting highly specific aptamers is not always achievable, however. For example, in some cases of cell-SELEX, which uses whole cells for selection, the resultant aptamers recognize both membrane proteins and membrane lipids [123]. As discussed in more detail later, selecting for targeting IDPs may also result in aptamers that cross-react with different structures of a targeted protein. Although determining aptamer specificity is a crucial step in characterizing aptamers, aptamer characterization has rarely been fully considered, especially for aptamers selected for cell-membrane targets [123] and for IDPs such as prion proteins (PrP) [124] or Aβ [125,126,127]. In the following two sections, I discuss why characterizing aptamer specificity is important in research into Aβ and, by extension, other IDPs.

## 4. Aptamers and Aβ

Aptamer studies using IDPs and amyloidogenic proteins so far show a general tendency for aptamers (and unselected, naïve oligonucleotide libraries) to preferentially bind to β-sheet-rich fibrillar amyloid assemblies despite selection against prefibrillar/nonfibrillar assemblies. For example, several groups have reported aptamers that bind PrP sequences [124,128,129,130,131,132,133,134]. An RNA aptamer selected for the recombinant bovine PrP reportedly recognized bovine PrP-β [134]—a soluble, oligomeric, β-sheet-rich conformational variant of full-length PrP that forms amyloid fibrils [135]. Bunka et al. generated aptamers for monomeric and several forms of fibrillar β2-microglobulin [136]. These aptamers were found to bind also fibrils of other amyloidogenic proteins, including apomyoglobin, Aβ40, transthyretin, or lysozyme, in addition to those of β2-microglobulin [136]. In the latter study, the naïve library also apparently reacted with long, straight fibrils of β2-microglobulin with half the strength of the selected aptamers [136]. Aptamers for α-synuclein have been reported and shown to bind strongly to α-synuclein oligomers but weakly to its fibrils [137,138]. Similar outcomes have been obtained in the context of Aβ as discussed in detail below.

The first study that described RNA aptamers for Aβ used a chemically synthesized monomeric Aβ40 preparation with an additional engineered N-terminal cysteine as SELEX target. This preparation was immobilized on a thiopropyl-activated Sepharose 6B matrix by disulfide bonding [125]. Importantly, because Aβ tends to aggregate rapidly, the authors coupled the Aβ40 preparation to Sepharose using 60% 1,1,1,3,3,3-hexafluoro-2-propanol (HFIP) in 10 mM Tris-HCl, pH 7.7, to keep Aβ40 disaggregated and soluble. (HFIP is used to dissociate self-assembling amyloid proteins [139,140].) The authors used a random 70-nucleotide RNA library (~10^15^ sequences) plus the flanking 5′ and 3′ primer sites. The library was first precleared (negative SELEX) using unloaded Sepharose and then incubated with Sepharose-bound Aβ40 at 4 µM on the resin. After washing the unbound RNA pool, the bound RNA was eluted with Aβ40 by dithiothreitol reduction of the disulfide bond. RNA was extracted, reverse transcribed to DNA, and amplified by PCR. After eight rounds of selection, ~140 binding sequences were eluted. The aptamers were then characterized by affinity chromatography to measure their dissociation constants, which ranged from 29 to 48 nM. Surprisingly, the selected aptamers did not bind soluble Aβ40 as tested by counter-elution using soluble Aβ40 and by mobility-shift assays. The aptamers showed unexpected binding to fibrillar assemblies of Aβ40 as observed by streptavidin–biotin conjugation, gold labeling, and electron microscopy. The authors concluded that Aβ40 may have aggregated on the matrix despite their using HFIP during Aβ40 conjugation to Sepharose [125], and thus selected aptamers bound fibrils nonspecifically.

As another example of aptamers selected for Aβ preparations, aptamers reported by Takahashi et al. so far are the only ones displaying binding affinity to an oligomeric “model” of Aβ40 [126]. The library pool in their study was incubated with Aβ40 conjugated to colloidal gold nanoparticles (10 nm diameter) acting as an “Aβ oligomer model,” which was described previously [20]. Two aptamers, N2 and E2, could bind this Aβ40 preparation when incubated at 4 °C and recognized Aβ40 in solution by fluorescence anisotropy. *K*_d_ values calculated from fluorescence anisotropy studies ranged from 11 to 22 µM. However, upon aptamer binding saturation with ~50 µM Aβ40, fluorescence anisotropy showed a change of 0.006–0.008 units which may well fall within the noise of such experiments (as discussed elsewhere [141]) despite the authors’ argument that this change may have resulted from the small mass of Aβ40. Thus, the reported *K*_d_ values remain questionable.

Conjugating Aβ40 to gold nanoparticles was first used to imitate spherical oligomers as antigen for generating the oligomer-specific antibody A11 [20]. A11 was found to react specifically with certain oligomeric preparations of Aβ40 and Aβ42 but not soluble, low-molecular-weight Aβ or fibrillar Aβ preparations [20]. Low-molecular-weight Aβ preparations comprise soluble, monomeric Aβ in dynamic equilibrium with low-order Aβ homooligomers [142]. Although arranging Aβ40 monomers on the surface of gold nanoparticles likely mimics high-order Aβ assemblies, and N2 and E2 aptamers likely preferably bound these structures, the ultimate proof of specificity is to exclude cross-reactivity of N2 or E2 aptamers with Aβ fibrils or fibrillar assemblies of other amyloidogenic proteins because of reported cross-reactivity of some “oligomer-specific” antibodies and “oligomer-specific” aptamers with fibrillar amyloid structures [21]. N2 and E2 were not tested for their cross-reactivity with fibrillar assemblies of Aβ or of other amyloidogenic proteins. They were not tested against other oligomeric preparations of Aβ or oligomeric preparations of other IDPs either. Similar to the case of N2 and E2, the aptamer M5-15, selected for the amyloidogenic protein, α-synuclein, reportedly reacted with monomeric and oligomeric forms of the target protein, but its cross-reactivity with α-synuclein fibrils was not tested [138].

Although N2 and E2 aptamers were not tested for their cross-reactivity with fibrillar assemblies of Aβ40 or Aβ42, they reportedly inhibited Aβ fibrillization as observed by ELISA using the 6E10 antibody [126]. (6E10 is a monoclonal antibody raised against residues 1–17 of human Aβ [143,144,145]). However, the reported ELISA results are surprising because of the following two caveats. First, at the initial time point, 6E10 ELISA did not detect the Aβ preparation either in the absence or in presence of the two aptamers, contradicting the fact that 6E10 reportedly reacts with random-coil (or statistical-coil) Aβ monomers [19,146]. Thus, at the initial time points, the sample without aptamers should have presented an ELISA signal at least as intense as that with the fibrillar preparation without added aptamers. Secondly, the authors did not exclude the possibility that the aptamers could compete with 6E10 binding to Aβ under the ELISA conditions. Thus, ELISA results may merely indicate low binding of the 6E10 antibody to the protein–aptamer mixture because of potential competition between aptamer and 6E10 for binding to fibrillar Aβ. Nevertheless, Aβ fibrils were not detected by electron microscopy in the presence of the aptamers; authors reported oligomers, protofibrils, and amorphous aggregates as potential products of fibril disintegration in the presence of aptamers [126]. Whether the abovementioned nonfibrillar Aβ assemblies were cytotoxic or not was not tested. Thus, the full reactivity/specificity spectrum and functions of these aptamers are yet to be confirmed.

Three years later, the N2 aptamer was reported as a conjugate to poly(lactic-*co*-glycolic acid)–coated curcumin (PLGA–curcumin) nanoparticles [147]. Aptamer–PLGA–curcumin nanoparticles were not cytotoxic, taken up by cells, and found to bind Aβ42 fibrils and disintegrate them [147]. Whether the fibril-disintegration products under such experimental conditions were cytotoxic or not was not tested, but the authors concluded that the fibril-degrading effect of curcumin was unaffected by conjugation of the aptamer to the PLGA–curcumin nanoparticles. The authors postulated that the resultant smaller amyloid fragments could easily be cleared by phagocytosis [147]. Interestingly, a recently published review [148] cites the above study, “… the N2 aptamer conjugated to curcumin-polymer nanoparticles enhanced binding to, and disaggregated, amyloid plaques, which were then cleared by phagocytosis”, misleading the reader by misreporting that actual “amyloid plaques” were used and “phagocytosis assays” were done in the original study [147]. Taking both studies [126,147] together, it is unclear whether the N2 aptamer or curcumin or both could bind Aβ42 fibrils (not plaques as mentioned [148], which are the in vivo hallmarks of AD) and degrade them because both activities were seemingly attributed to curcumin and N2. Importantly, curcumin along with resveratrol and epigallocatechin-3-gallate (reviewed [149]) have been dubbed as pan-assay-interfering compounds [150,151], and conclusions made about these three polyphenols in the AD literature in relation to their effects on Aβ should be reassessed carefully [152].

We asked why aptamers selected for monomeric or prefibrillar assemblies of amyloidogenic proteins recognized their polymeric, fibrillar forms. Could specific aptamers for monomeric and/or oligomeric forms of an amyloidogenic protein ever be obtained? What are the implications of fibril reactivity of RNA or DNA aptamers? To answer these questions, we performed SELEX to obtain aptamers that could potentially recognize the covalently stabilized trimeric Aβ40 [153], which were produced by using photo-induced crosslinking of unmodified proteins (PICUP) [154,155], extracted from gels subjected to SDS–PAGE, and purified by removing SDS [156]. We also used a mixture of low-molecular-weight oligomeric Aβ40, which was generated by PICUP but not exposed to SDS at all, in later experiments. (The significance of SDS effects on Aβ preparations is discussed below).

I summarize the main findings of that study: (1) aptamers selected for purified, covalently stabilized trimeric Aβ40 failed to react with purified Aβ40 trimers or with the low-molecular-weight mixture of prefibrillar Aβ40 assemblies, but they reacted with Aβ40 or Aβ42 fibrils, as confirmed by dot blotting. (2) Aptamers selected for recognizing trimeric Aβ40 reacted not only with Aβ fibrils, but also with fibrils of other amyloidogenic proteins, including calcitonin, islet amyloid polypeptide (IAPP), insulin, lysozyme, and prion_106–126_. (3) Our aptamers reacted with fibrils of the tested amyloidogenic proteins similarly to β aptamers selected for Aβ40 previously [125] and reused/retested for imaging Aβ plaques [127]. (4) To exclude the possibility of SDS contamination in our trimeric Aβ40 preparation, we used a PICUP-generated mixture of low-molecular-weight Aβ40 preparation, which was not subjected to SDS–PAGE. As discussed below, SDS is known to accelerate Aβ self-assembly and β-sheet formation [157]. In these series of SELEX experiments, we included two counter-SELEX cycles against Aβ40 fibrils after the fourth and fifth SELEX cycles. The RNA pool obtained after the fifth SELEX cycle reacted with fibrils of Aβ and fibrils of the other tested amyloidogenic proteins similarly to our aptamers [153] and β aptamers selected previously [125]. This finding indicated that counter-selection against Aβ40 fibrils could not effectively remove aptamer reactivity with fibrils. (5) Because of this finding, we performed another SELEX cycle with several counter-SELEX experiments using Aβ40 fibrils aiming to obtain an RNA pool devoid of fibril-binding sequences. However, five consecutive rounds of counter-SELEX using excess Aβ40 fibrils failed to reduce the binding of the RNA pool to Aβ40 fibrils. (6) Because of the persistent and apparently non-specific binding of RNA aptamers to amyloid fibrils, and because counter-SELEX using Aβ40 fibrils failed to abrogate aptamer binding to amyloid fibrils, we assessed our naïve RNA library and a G-biased RNA library for their reactivity with amyloid fibrils. We used the biased library with reduced G ratio (A:C:G:T = 30%:30%:10%:30%) because our sequencing and motif analyses showed high G content in selected aptamers. We found that both naïve RNA libraries reacted with fibrillar assemblies of the same proteins akin to all the selected aptamers we tested [153]. (7) The selected aptamers—and the naïve library—could track progression of β-sheet formation and fibrillization in Aβ40 and insulin with ~16-fold higher sensitively than the thioflavin T fluorescence assay, which is commonly used to assess fibril formation by many amyloidogenic proteins [158,159]. (8) HFIP-treated lysozyme and IAPP contained sufficient β-sheet content as inferred from their recognition by the tested aptamers and the naïve library. Our observation of non-specific reactivity with fibrils of selected and tested aptamers, which is reminiscent of similar findings in previous studies [62,125,134,136], suggest that aptamers (and naïve libraries of oligonucleotides) likely recognize potentially common aptatopes [62,153,160].

Studies that selected aptamers for recognizing Aβ assemblies are summarized in Table 1.

Farrar et al. [127] used the β55 aptamer, which was published [125] and retested [153] previously, for ex vivo imaging of frozen sections of human AD brain fixed in paraformaldehyde, while including the corresponding reverse sequence of β55 as control. The authors performed in vivo multiphoton microscopy using the APP–PS1 transgenic mouse model of AD [161] to visualize plaques [127]. Biotinylated β55 reportedly stained many more plaques than its reverse sequence, and β55 staining localized with thioflavin-S signal, confirming staining of amyloid plaques ex vivo [127], and by inference confirming binding to Aβ fibrils as shown by previous studies [125,160]. Fluorescein-conjugated β55 stained amyloid plaques and amyloid angiopathic lesions in brains of APP–PS1 mice visualized by multiphoton microscopy [127]. In localization staining experiments, β55 and methoxy-X04 stained the dense core of the plaques, whereas β55 additionally stained a diffuse halo surrounding the plaque cores [127]. (Methoxy-X04 is a derivative of Congo Red and it has been used previously for optical imaging of AD mouse models [162]. Congo Red is used to stain and detect amyloid depositions in tissues. Upon binding to amyloid structures, Congo Red yields a unique blue–green birefringence under a cross-polarized light microscope [159].) Farrar et al. [127] concluded that β55 may have bound smaller aggregates, including oligomers, of Aβ peripheral to the dense plaques based on their observation that β55 apparently bound low-molecular-weight oligomers of Aβ40 and Aβ42 on SDS–PAGE gels, and similar observation reported by Koffie et al. [163], showing a “halo of oligomers” surrounding the plaques detected by a so-called “conformation-specific” NAB61 antibody [164]. However, Farrar et al. [127] did not test nor compared the sensitivity of methoxy-X04 with that of β55 for binding small, early, β-sheet-containing fibrillar aggregates of Aβ. Possibly, methoxy-X04 could not sensitively detect the small β-sheet-containing fibrillar Aβ similarly to thioflavin T, which failed to detect early, sparse Aβ40 and insulin fibrils, but RNA aptamers detected early fibrillar assemblies of Aβ40 and insulin containing β-sheet structure [153]. Implications of β55 aptamer binding to SDS-fractionated Aβ species and Farrar’s conclusions about β55’s ability to detect oligomeric Aβ species around Aβ plaques are discussed in more detail in the following section.

## 5. SDS–PAGE, Aptamers, Antibodies, and “Halos of Oligomers”

Along with silver staining, Coomassie staining, western blotting, or mass spectrometry, SDS–PAGE has been used to identify proteins and examine protein oligomerization, size distribution, or protein–protein interactions. However, SDS (288.38 g·mol^−1^) does not affect all proteins identically [165] because different proteins, different conformations of a protein [166], or fragments of certain proteins [167] may not bind SDS at stoichiometric amounts (though SDS generally binds different proteins at an approximately constant mass–mass ratio—1.4 g SDS per gram of polypeptide [159]). Furthermore, in certain cases, SDS induces or stabilizes—rather than disrupting—secondary or quaternary structures [166,168,169]. In some other cases, SDS may induce homo-oligomerization or conversely dissociate protein complexes [169,170,171,172]. For example, both human and rat α-synuclein show aberrant electrophoretic mobility and SDS–PAGE-induced high-molecular-mass components, which do not exist in the samples when analyzed by size-exclusion chromatography [173].

Aβ is an amphipathic protein that forms “SDS-stable oligomers” [174,175]. In fact, SDS-induced aggregation of Aβ has facilitated extraction of Aβ from brain homogenates [176]. Aβ42-derived “globulomers” are in vitro model oligomeric species produced by incubating Aβ42 with 0.2% SDS [177,178]. Aβ aggregates rapidly after being treated with SDS and forms high-molecular-mass assemblies [157]. During electrophoresis of Aβ40, its SDS-induced aggregates dissociate and only a monomeric component is observed by staining, whereas electrophoresis of Aβ42 yields apparently trimeric and tetrameric components as observed previously [179,180]. In addition, essentially identical monomer–trimer–tetramer components appear when different Aβ42 preparations, including monomeric, oligomeric, or fibrillar Aβ42, are subjected to SDS–PAGE [181], demonstrating that SDS treatment, and electrophoresis in the presence of SDS—rather than the initial assembly state—determines Aβ42’s apparent PAGE mobility. After treatment in a urea-containing SDS–PAGE system, Aβ and its truncated versions defy the mass–mobility relationships, because Aβ–SDS interaction likely does not relate to the number of constituent amino acids but to the sum of hydrophobicity indices [167]. An exemplary study of Aβ40 dimers stabilized by an intermolecular disulfide bridge showed the same SDS–PAGE profile before and after formation of β-sheet-rich Aβ protofibrils [182]. Watt et al. compared SDS–PAGE, the xMAP^®^ multiplex immunoassay (Luminex, Madison, WI, USA) and surface-enhanced laser desorption/ionization time-of-flight mass spectrometry when examining Aβ extracted from human cortical tissues [183]. Their mass-spectrometry experiments could not detect oligomers, while monomeric and dimeric Aβ components appeared through SDS–PAGE; surprisingly, the apparent monomeric and dimeric Aβ levels increased with increasing SDS concentrations in the sample buffer [183]. Thus, electrophoretic separation and detection of monomeric or oligomeric assemblies in an Aβ preparation do not necessarily prove that such components exist in the sample before SDS–PAGE.

The shortcomings of SDS–PAGE have been highlighted in many studies of Aβ [21,60,157,159,179,181,183] and α-synuclein [173] and is gradually being appreciated in the AD field. Meanwhile, interpretations of findings about elusive Aβ oligomers has come under scrutiny and disputed to such an extent that the foundations of the oligomer cascade hypothesis have been shaken. Accordingly, a relatively recent study has critically evaluated the use of SDS–PAGE, claiming that the concept of Aβ oligomers has disserved decades of research into AD [184]. This study used ion mobility coupled with electrospray-ionization mass spectrometry (ESI–IM–MS), challenging the biophysical paradigms dominating the Aβ field based on SDS–PAGE and PICUP analyses of prefibrillar assemblies of Aβ. When coupled with MS, ion-mobility spectrometry, which distinguishes ions according to both their mass-to-charge ratio and their three-dimensional structures, is a useful analytical technique for examining covalent or non-covalent protein structures in complex mixtures. Ion-mobility spectrometry–mass spectrometer (IMS–MS) can resolve molecules of identical mass-to-charge ratios with differing collision cross-sections (e.g., different assembly states or conformations) and/or differing charge states [159]. Pujol-Pina et al. used PICUP-stabilized Aβ oligomers and showed that the Aβ42 pentamer–hexamer components observed by SDS–PAGE following PICUP are methodological artifacts [184]. The authors removed the SDS–PAGE step from analyzing PICUP-generated, cross-linked Aβ40 and Aβ42 preparations and, instead, used size-exclusion chromatography and ESI–IM–MS. Since initial PICUP–SDS–PAGE observations, Aβ40 and Aβ42 were thought to oligomerize and aggregate through distinct pathways [154,179]; that is, Aβ42 was thought to aggregate through formation of “paranuclei”—the pentamer–hexamer subunits—and distinctly from Aβ40, which was thought to aggregate through dimer–trimer–tetramer subunits. By excluding SDS–PAGE, ESI–IM–MS showed no differences in the oligomer-size distribution between cross-linked or uncross-linked Aβ40 and Aβ42, suggesting that Aβ40 and Aβ42 predominantly and similarly initiate oligomerization and aggregation through dimer–trimer subunits [184]. The implications of the ESI–IM–MS findings controverts the conclusions that C-terminal length of Aβ was the most important structural determinant in early oligomerization, and the side-chains of Ile41 and Ala42 in Aβ42 were important both for effective formation of paranuclei and for their self-association [179,185]. It was discussed previously that differences in toxicity between Aβ40 and Aβ42 [186] correlate with PICUP–SDS–PAGE observations that paranuclei are produced by Aβ42 only, confirming the correlation of the latter to AD pathogenesis.

As another example, the elaborate study by Koffie et al., which used ultrathin array tomography and immunofluorescence, claimed that senile plaques in brains of AD model mice are surrounded by “haloes of oligomeric Aβ” [163]. This conclusion was mainly based on immunoreactivity of NAB61, which apparently reacted with oligomeric Aβ assemblies fractionated by SDS–PAGE [164]. The original paper, which described this antibody as a “Conformation-selective Monoclonal Antibody,” ironically reported that NAB61 also recognized synthetic Aβ fibrils by electron microscopy, as presented in its small Figure 4B panel [164]. Considering these caveats, one may rightly question the major conclusions drawn by Koffie et al. [163], and the same interpretative analogies repeated and drawn by Farrar et al. [127]. The former used NAB61 and the latter β55 and claimed that the antibody and aptamer were specific for SDS–PAGE-fractionated oligomeric Aβ and, in this capacity, detected Aβ oligomers around plaques, ignoring the shortcomings of SDS–PAGE (firstly) and the fact that β55 and NAB61 *both* cross-react with fibrillar Aβ assemblies besides SDS-fractionated Aβ species (secondly). Similar cross-reactivity was apparent in antibodies that were produced and characterized after iterative immunization of beagles [187] with an aggregated Aβ preparation [188]. Thus, the conclusions by Farrar et al. [127] about staining small oligomers haloing the dense plaques as observed by β55 must be reexamined in light of the collective literature regarding (1) SDS–PAGE analysis of Aβ; (2) NAB61 reactivity with Aβ assemblies; (3) plaque immunohistochemistry; (4) and sensitivity of the aptamer binding compared to methoxy-X04 (or thioflavin T/S) binding to Aβ fibrils—and plaques.

To sum up, despite its wide use and resolution, SDS–PAGE and western blotting are not reliable methods for determining oligomer sizes or assembly states of certain IDPs, e.g., α-synuclein and Aβ oligomers. As such, SDS–PAGE is not suitable for assessing the specificity or selectivity of aptamers (or antibodies) for Aβ preparations. Considering SDS–PAGE’s shortcomings is important for characterizing the reactivity and specificity of aptamers or antibodies generated against Aβ species (see [127,163,164]) because SDS-induced oligomers in an Aβ preparation are not necessarily structurally the same as those potentially present in the absence of SDS [184].

## 6. Conclusions

The conclusions from this review are manifold.

- The handful of reports published since 2002 on aptamers developed for targeting Aβ have led to important and instructive findings. RNA and DNA aptamers and random nucleotide libraries used for selecting aptamers are found to react inherently and nonspecifically with fibrillar Aβ preparations and exemplary amyloid assemblies [21,153,160]. Most likely, the aptamer-targeted common aptatope in these cases is the backbone of the proteins in a cross-β structure because this protein structure reportedly facilitates retention of RNAs and RNA-binding proteins into special ribonucleoprotein complexes, including stress granules and RNA-processing organelles [189]. The inherent and persistent tendency of RNA aptamers to bind amyloid fibrils (or vice versa) may explain entrapment of RNA in the senile plaques and neurofibrillary tangles [73,74,75], the two pathological hallmarks of AD brains. Moreover, amyloid fibrils and oligonucleotides act as polyelectrolytes and interact by electrostatic forces [190]. These β-sheet-mediated, polyelectrolytic, protein–oligonucleotide interactions were thought to be vital for support, stability, compartmentalization, protection, and resistance to degradation in the harsh environments of the antediluvian, prebiotic world [191], indicating an ancient phenomenon. Interaction of RNA aptamers with amyloid fibrils have implications for the previous and future studies of aptamers selected for amyloidogenic proteins and conclusions drawn from such studies.
- Attributing oligomer specificity to an aptamer based on results obtained by SDS–PAGE fractionation of Aβ preparations disregards the collected evidence on the unsuitability of SDS–PAGE for analyzing and size estimation of amyloidogenic protein assemblies.
- Attributing oligomer specificity to an aptamer (or an antibody) that evidently binds fibrillar structures of amyloidogenic proteins (see [127,163]) is erroneous and misleading; thus, binding specificities of such aptamers in tissue sections do not represent their true specificities and enhances the illusion about presence of Aβ oligomers in tissue sections.
- Implications of SDS–PAGE are extendable to studies whereby prefibrillar amyloid assemblies were extracted and studied in vitro [192,193,194,195,196,197,198,199] or PICUP-stabilized oligomers were studied to establish the biophysical paradigms of Aβ oligomerization [179,184,185].
- Finally, I hope this review could encourage the aptamer–amyloid–Alzheimer researchers, the relevant funding bodies, these fields’ peer-reviewers, and the fields’ young scholars to scrutinize and study the relevant literature deeply before enthusing [148,200,201,202] about aptamers in the context of Aβ research. Let us not generate an aptamer field akin to the muddled assortment of antibodies promoted in AD research [21,22].

## Figures and Tables

**Table 1:** Aptamers selected for interacting with different amyloid β-protein (Aβ) preparations.

Aptamer Type	Target	SELEX Method	Aptamer Reactivity	Reference
RNA, β aptamers, e.g., β55	Synthetic Aβ40 with an engineered N-terminal cysteine	Chromatographic separation using Sepharose 6B matrix carrying the target	No interaction with monomeric, soluble Aβ40, but reactive with Aβ40 fibrils	[125]
RNA aptamers E1, E2, N1, G2 etc.	Aβ40 conjugated to gold nanoparticles as a model of Aβ oligomers	RNA pool was exposed to target, separation was by centrifugation, and three different elution strategies used	Aβ40 oligomer model and apparently monomeric Aβ40	[126]
RNA aptamers, KM and previously reported β aptamers	PICUP-generated and purified trimeric Aβ40, and a PICUP-generated mixture of low-molecular-weight Aβ40 oligomers	Filter-binding assay used for separation	Aβ fibrils and fibrils of other exemplary amyloidogenic proteins	[153,160]

SELES, systematic evolution of ligands by exponential enrichment; Aβ, amyloid β-protein; PICUP, photo-induced crosslinking of unmodified proteins.

## Abbreviations

Aβ	amyloid β-protein
AD	Alzheimer disease
APP	amyloid β-protein precursor
ELISA	enzyme-linked immunosorbent assay
ESI–IM–MS	ion mobility coupled with electrospray-ionization mass spectrometry
HFIP	1,1,1,3,3,3-hexafluoro-2-propanol
IAPP	islet amyloid polypeptide
IDPs	intrinsically disordered proteins
IM–MS	ion-mobility spectrometry–mass spectrometery
PCR	polymerase chain reaction
PICUP	photo-induced crosslinking of unmodified proteins
PLGA	poly(lactic-*co*-glycolic acid)
PrP	prion proteins
SDS–PAGE	sodium dodecyl sulfate–polyacrylamide gel electrophoresis
SELEX	systematic evolution of ligands by exponential enrichment
